# Thinking on the Construction of Antimicrobial Peptide Databases: Powerful Tools for the Molecular Design and Screening

**DOI:** 10.3390/ijms24043134

**Published:** 2023-02-05

**Authors:** Kun Zhang, Da Teng, Ruoyu Mao, Na Yang, Ya Hao, Jianhua Wang

**Affiliations:** 1 Gene Engineering Laboratory, Feed Research Institute, Chinese Academy of Agricultural Sciences, Beijing 100081, China; 2 Innovative Team of Antimicrobial Peptides and Alternatives to Antibiotics, Feed Research Institute, Chinese Academy of Agricultural Sciences, Beijing 100081, China; 3 Key Laboratory of Feed Biotechnology, Ministry of Agriculture and Rural Affairs, Beijing 100081, China

**Keywords:** antimicrobial peptides, databases, characteristic function, screening model

## Abstract

With the accelerating growth of antimicrobial resistance (AMR), there is an urgent need for new antimicrobial agents with low or no AMR. Antimicrobial peptides (AMPs) have been extensively studied as alternatives to antibiotics (ATAs). Coupled with the new generation of high-throughput technology for AMP mining, the number of derivatives has increased dramatically, but manual running is time-consuming and laborious. Therefore, it is necessary to establish databases that combine computer algorithms to summarize, analyze, and design new AMPs. A number of AMP databases have already been established, such as the Antimicrobial Peptides Database (APD), the Collection of Antimicrobial Peptides (CAMP), the Database of Antimicrobial Activity and Structure of Peptides (DBAASP), and the Database of Antimicrobial Peptides (dbAMPs). These four AMP databases are comprehensive and are widely used. This review aims to cover the construction, evolution, characteristic function, prediction, and design of these four AMP databases. It also offers ideas for the improvement and application of these databases based on merging the various advantages of these four peptide libraries. This review promotes research and development into new AMPs and lays their foundation in the fields of druggability and clinical precision treatment.

## 1. Introduction

**Figure 1:**
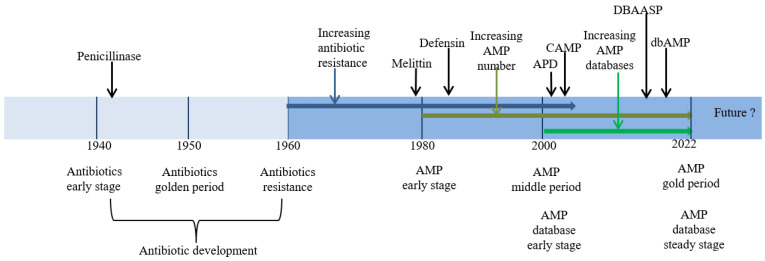
Timeline of research progress on antibiotics, antibiotic resistance, AMPs, and AMP databases.

Antibiotics represent one of the major discoveries made in the field of health during the 20th century. Starting with the discovery of penicillin in 1942 as the first key milestone, antibiotics have greatly benefited humanity, playing a key role in the treatment of human and animal diseases. However, due to the long-term abuse of antibiotics, especially in husbandry production, many bacteria have formed and have developed AMR over time. These bacteria include *Staphylococcus aureus*, *Streptococcus*, *Escherichia coli*, and other species. Some of them have developed multi-drug resistance quickly, which significantly reduces the efficacy of antibiotic treatment [1,2,3,4]. The first sulfonamide drug with a special resistance mechanism was reported in 1937, but the threat of AMR received little attention at that time. After drug-resistant plasmids were first reported in 1960, the number of antimicrobial-resistant bacteria steadily increased year by year in the nearly 30 years that followed [1,5]. There is now an urgent need for a series of new ATAs to address this issue. Figure 1 shows the timeline of resistance development for the major classes of antibiotics.

**Figure 2:**
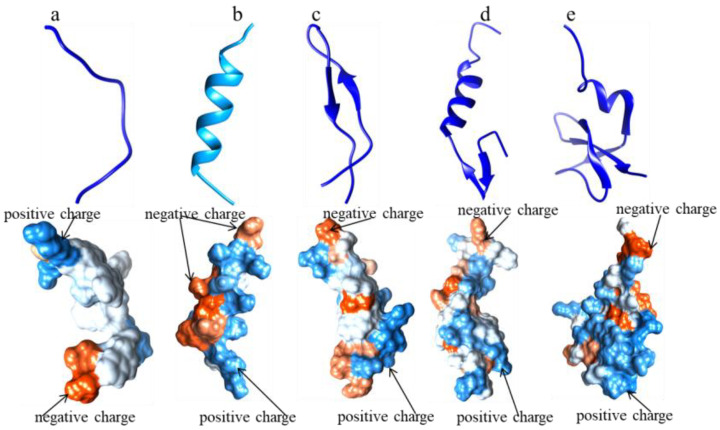
The 3D structures of typical AMPs (Source: Protein Data Bank (PDB); Tool: UCSF Chimera). (**a**) 1F0F: AMP, the α-helix structure of CecropinA (1–8); (**b**) 1LFC: AMP, the β-sheeted structure of LactoferricinB; (**c**) 1CW6: AMP, α-helix and β-sheeted unpacked structure of LeucocinA; (**d**) 1KJ6: AMP, the α-helix and β-sheeted packed structure of hBD3; (**e**) 1g89: AMP, the structure line of 1g89.

AMPs are produced naturally in organisms and act as an innate defense system against invading pathogens via diverse mechanisms of action [6]. Melittin and maganin were first discovered by Fennell and Zasloff in 1967 and 1987, respectively [7,8]. In Sweden, Boman’s team discovered and reported typical antibacterial peptides known as cecropins from the insect Hyatophoraceropia during the 1970s and 1980s [9,10,11,12,13], marking a key moment in the development of AMP science. From 1980 to 2000, AMPs, including defensins, cecropins, and magainin, were isolated from humans, insects, and marine animals. Since then, the number of AMPs has increased dramatically, accelerating the establishment and development of AMP databases [14,15,16,17,18,19]. AMPs are naturally produced by humans, animals, bacteria, and fungi and include bacteriocins and fungus defensins. They are all involved in antimicrobial and immune regulation at trace levels and respond in vivo [14,15,18,20,21,22,23]. In addition, AMPs are also reported to have anticancer, antiviral, antiparasitic, and antibiofilm functions [24,25,26]. For example, Maximin-1, Dermaseptin-B2, Macropin-1, HBD-3, and Opis were discovered in 2002, 2010, 2012, 2017, and 2020, respectively, and have antiviral, antimicrobial, antifungal, and antiparasitic properties [18,20,23,25]. On a basis of works over the past two decades, the “iron triangle” theory for the prevention and treatment of human and animal diseases has been recently proposed serving the One Health concept, consisting of AMPs, antibiotics, and vaccines and focusing on strong penetration, high internalization, and low AMR [26,27,28,29,30,31]. Most studies have emphasized that a better understanding of the structure and activity of peptides is vital and have demonstrated the value of databases to classify them. In terms of research, application, and construction, it is known that most AMPs, with a length of about 50 amino acids, have cation characteristics (+6~+8) and can have both hydrophobicity and amphiphilicity [17,23,24,25]. Their structures include α-helix, β-sheet, linear, and α-helix and β-sheet combinations. For example, CecropinA, LactoferricinB, LeucocinA, HBD-3, and indolicidin have α-helix, β-sheet, α-helix plus β-sheet unpackaged, α-helix plus β-sheet packaged, and linear structures, respectively [32,33,34,35,36]. The structure and physicochemical properties of these AMPs are summarized in Figure 2. Around the year 2000, some AMP databases were constructed according to the charge, length, antimicrobial activity, and structure of AMPs and mainly functioned as prediction tools based on the natural templates of AMPs. The key amino acids as cysteine, lysine, arginine, and glycine, among others, in the sequences of AMPs significantly affect their structure and physical properties, especially the status of cationic and hydrophobic properties [37,38,39,40,41,42,43]. However, there is a threshold beyond which strong hemolysis and cytotoxicity can follow [44,45]. Most AMPs interact with bacterial anionic lipid membranes or viral capsids through cationic attraction and hydrophobicity through processes such as the carpet, β-barrel wall, or ring pore models [46,47]. They further combine with nucleic acids, intracellular proteins, and enzymes to inhibit transcription, translation, and biosynthesis, thereby inhibiting the formation of cell walls, cell membranes, or even the cell cycle. Studies have shown that AMPs such as those in the defensin family target the binding of bacterial lipids to exert high antibacterial effects [48,49,50,51]. With the rapid increase in the number of AMPs, the processes of in vivo/vitro, one-by-one, and step-by-step verification use a number of resources in terms of design, screening, and confirmation. Most AMPs suffer serious limitations with regard to low yield, instability, and toxicity. Therefore, it is necessary to establish an AMP database and to combine it with computer algorithms to efficiently and accurately predict and design new AMPs [52,53] and to further validate the iron triangle theory [26,27,28,29,30,31] and its application in health maintenance.

More than ten AMP databases have been established to collect and classify AMPs so far, including APD3, DBAASPv3, CAMP3, dbAMP2, ANTI- MIC, YADAMP, LAMP2, DRAMP3.0, CyBase, and PenBese [54,55,56,57,58,59]. Among them, the first four are the most popular because of their superior tool buffering, large data resources, and powerful function, thus attracting more users [60]. These four databases were first built in 2005, 2008, 2014, and 2018 [56,60,61,62,63,64] and updated in 2016, 2016, 2021, and 2022, respectively [55,65,66,67]. The data resources and analytical functions of AMP databases are their essential features. Now, more and more AMP databases are being recognized as bioinformatics resources to identify, predict, and design new AMP derivatives with better or improved properties. For example, non-hemolytic anti-MRSA AMPs from plant sources have been obtained using the above tools to design them [42,56,68]. Although a variety of AMP databases have been established, they have not been applied fully or extensively, due to their weak reliability for prediction ability in design processes [60]. Only data acquisition and prediction are used in practice. Further resources are urgently needed to support additional requirements such as AMP mining [68], DNA editing, AMP AI editing [69], complex BI analysis [70], computer-aided design [71], and chemical and synthetic biology [72,73,74,75]. When considering how to supplement these disadvantages in AMPs and achieving the above goals in AMP science in the future, there is room for improvement. There are large challenges facing meeting the above new requirements for AMPs in health practices in humans and animals. The evolution of antibiotics, AMPs, and AMP databases is shown in the timeline in Figure 1.

In this paper, the advantages, disadvantages, applications, and challenges associated with four AMP databases are reviewed, and some suggestions for the construction of databases carrying out quick screening and exact predictions are provided. Focusing on design running and on the basis of four typical AMP databases, key principles resulting in increased and better advantages and stronger tool power are put forward to create a new scheme.

## 2. Four Typical AMP Databases

**Figure 3:**
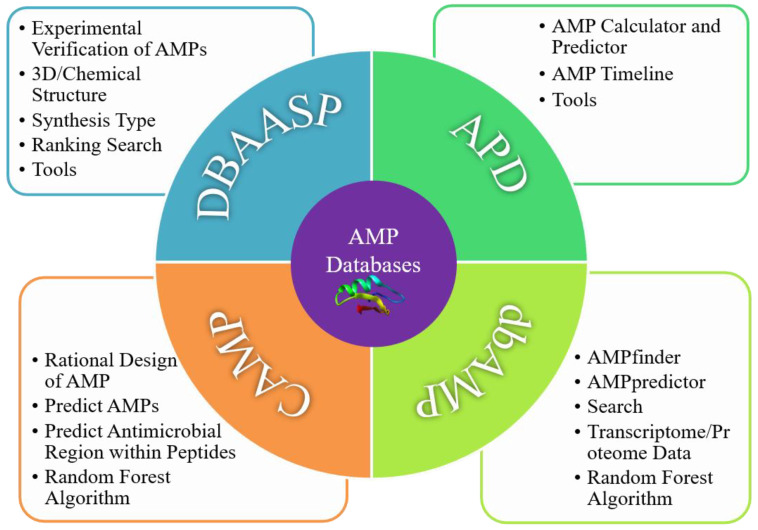
Advantageous modules of four AMP libraries.

AMP databases usually feature a number of functions, such as large datasets with logistical classification, accurate prediction abilities, fast searching, and unique computer algorithms. Their most important features include prediction tools and abundant data from different pathways. Those prediction tools were developed by analyzing the physicochemical properties, toxicity, and specificity of AMPs. Four databases (DBAASP, CAMP, APD, and dbAMP) are the most popular so far (see Table 1 and Table 2); their advantageous modules are shown in Figure 3. They are introduced one by one in the following sections [61].

### 2.1. DBAASP

DBAASP is a database that is curated manually that collects experimentally validated AMPs through experiments in which the physicochemical properties can be predicted or analyzed [56]. Recently, the 3D structures of the AMPs in this database were updated [63]. Presently, a total of 18,719 entries have been collected and classified in DBAASP (Table 1 and Table 2) [66]. It is the most comprehensive database for evaluating the antimicrobial activity, cytotoxicity, and hemolysis of target peptides obtained through the collection of validated AMPs from laboratory studies. Users can search by peptide ID, name, synthesis type, sequence, length, C-terminal N-terminal modification, family source, intracellular target, UniProt ID, BD structure, hemolysis, and others fields to obtain the target sequence. Another advantage of DBAASP compared to other databases is its capacity to learn the structural and functional relationships of AMPs (Figure 3). Of course, instability, molecular weight, secondary structure, and half-life parameters should be added or supplemented if possible, and more machine learning (ML) algorithms should be adopted to increase and ensure the accuracy of prediction results.

### 2.2. APD

The APD database was established in 2003 by Wang Guangshun team and has been updated in recent years. It contains 1228 peptides (including 65 anticancer peptides, 76 antiviral peptides, 327 antifungal peptides, and 994 antibacterial peptides) and offers search capability [76], statistical analysis, structure–function relationships, and other AMP indexes [66]. Currently, there are 3425 AMPs in the APD3 database, which are mainly derived from natural species and are very close to the actual number of reported active AMPs with high reliability (Table 1 and Table 2). Through this database, the physical and chemical properties of AMPs, including their molecular size, isoelectric point, hydrophilicity, structure, hydrophobic residues, protein-binding capacity, and net charge, can be predicted and calculated. Another feature is the AMP timeline module, allowing a better understanding of AMPs in relation to time (Figure 3). This database is considered to be the best tool for learning about the development and predicting the physical and chemical properties in AMPs. The APD3 database is in need of improvement. Its capacity for buffering candidates and physicochemical properties is relatively limited: for example, some derived peptides with better antibacterial activity are not included in the library or are not classified in detail. Furthermore, the in-depth analysis of anti-Gram-negative/positive bacterial peptides, more family sources, and better ability to predict on the potential and toxicity of AMPs should be integrated.

### 2.3. CAMP

The Collection of Antimicrobial Peptides (CAMP), established by Shaini Thomas in 2010, is a free online database that includes mature ML algorithms for various AMPs, initially including 3782 AMPs: 2766 AMPs from experimentally verified patents/non-patents and 1016 predicted sequences [62]. Its latest version features 10,247 sequences, containing 8164 AMPs, 2083 patented AMPs, 757 structures, and 114 AMPs with family-specific features (Table 1 and Table 2). The best feature of CAMP is its prediction tools based on ML algorithms such as Random Forest (RF), Support Vector Machine (SVM), and Discriminant Analysis (DA), achieving accuracy levels of 93.2%, 91.5%, and 87.5%, respectively. This database marks the relationship between sequence structure and antibacterial activity for the first time and is useful for searching sequence activities and for determining their specificity and relationships with AMPs [77,78]. By analyzing sequence signatures consisting of patterns and Hidden Markov Models (HMMs) from 1386 experimentally studied AMPs, 45 AMP families have been generated in this database. It is expected that sequence optimization algorithms to rationally design amplifiers will be widely used (Figure 3) in design practices in the future. In addition, regarding the physicochemical properties of AMPs, such as hydrophobicity, net charge, instability, amphipathicity, and toxicity, the statistical results and derived peptides should be further improved.

### 2.4. dbAMP

The dbAMP database is the largest database and was developed by Tzong-Yi Lee in 2018 [64]. It initially contained 12,389 AMPs able to be retrieved through the NCBI, UniProt, PDB, and AMP databases, such as APD3, CAMPR3, ADAM, PhytAMP, AMPer, Antip2, BACTIBASE, and LAMP. References can be retrieved by querying the searchable fields of AMP-related articles individually [64]. The latest version, updated in 2022, includes 26,447 AMPs and 2262 antimicrobial proteins, with 4579 references [79] (Table 1 and Table 2). It also offers transcriptomic and proteomic data from all species quickly and simulates the 3D structures of AMPs online. Thus far, a total of 458 3D-structured AMPs have been collected and are available to users [65]. Compared with other databases, its best feature is the capacity to predict the activity of AMPs on different target bacteria, viruses, cancer cells, fungi, and mammals and to handle the transcriptomic and proteomic data obtained by applying high-pass technologies such as mass spectrometry (Figure 3). Because of this, it has particular value when dealing with transcriptomic and proteomic data and when analyzing their specificity. In addition, AMPs can be searched by their dbAMP ID number, although this feature works less smoothly than is desirable. Another negative is that the dbAMP database lacks the ability to predict physicochemical properties such as the hydrophobicity, net charge, amphiphilicity, instability, and hemolysis of AMPs.

## 3. ML Methods of the Four AMP Databases

**Figure 4:**
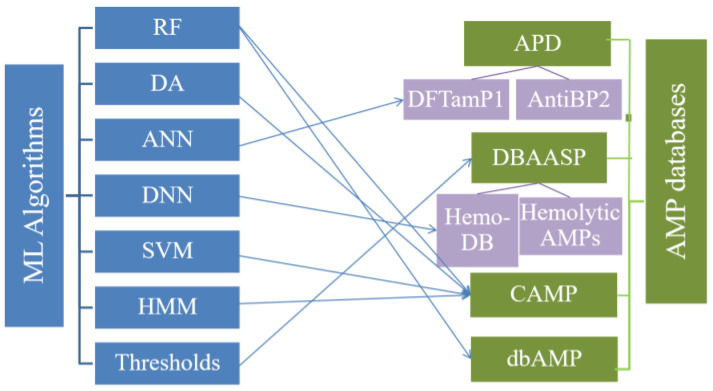
AMP Databases and ML algorithms.

Machine learning (ML) algorithms have been integrated and used in a variety of disciplines such as psychology, biology, and neurophysiology as well as in mathematics and automation. They can improve the production of vaccines and the design and screening of AMPs and target drugs to improve efficiency and to reduce drug application [80]. The combination of biology and ML has greatly promoted the development of bioinformatics, in which many amino acid sequences of AMPs with higher-complexity structures are analyzed quickly, especially when processing high-throughput data from transcriptomics and proteomics [80,81]. At present, some mature ML algorithms are used in prediction software to categorize and analyze data, and the newly developed AMP databases also contain classical machine algorithms such as RF, SVM, DA, Artificial Neural Network (ANN), and Deep Neural Network (DNN) [82,83,84]. Previous studies have shown that MLs are an important feature of databases, especially in the CAMP database, which contains all of the above MLs algorithms for the prediction and design of AMPs [77], whereas only parameter spaces and thresholds or cut-off discriminator algorithms are embedded in the APD and DBAASP databases, respectively [66,67]. Many specific AMP databases, such as those for linear cationic AMPs (LCAP), hemolytic and non-hemolytic AMPs, and anti-Gram-negative peptides (PHNX), combining ML algorithms have been established [85,86,87,88,89,90]. For example, the ML algorithms integrated with ANtiBP2, Hemdytik, and DASamp1 are called ANN and DNN [85,86]. The ML algorithms implemented by the four AMP databases analyzed here, and their derived databases, are summarized in Figure 4.

## 4. Challenges Facing the Application of Four AMP Databases

Due to the rapid development of AMP databases, they are being widely used in many fields, with two notable highlights being observed: the abundant data resource and the prediction and design of AMPs. ML algorithms are being involved in these processes, promoting deep learning on AMPs [86].

### 4.1. Application of APD and CAMP

These two databases have been widely used to design new AMPs of anti-methicillin- resistant *Staphylococcus aureus*, hemolytic and non-hemolytic AMPs, and anti-*HIV*-1 peptides [91,92,93,94,95] and include AMPs of anti-*Acinetobacter baumannii*, anti-*HIV*-1, cysteine-free AMPs, and cuttlefish AMPs [93,94,95]. Combining the two databases helps to design and screen special AMPs. Houyvet reported using APD3 and CAMPR3 from these databases to obtain nine AMPs with a length of less than 25 amino acids from cuttlefish (*Sepia officinalis*) [95].

### 4.2. Application of DBAASP

The hemolytic property of AMPs is one of the major obstacles hindering their clinical application [85]; therefore, it is essential to select special characteristics with low hemolytic targets. The DBAASP database has been used to design non-hemolytic AMPs of anti-methicillin-resistant *Staphylococcus aureus* (MRSA). Capecchi designed special AMPs using DBAASP and non-hemolytic AMPs using RNN in 2021; a total of 28 AMPs were synthesized and tested, and a final total of eight novel non-hemolytic AMPs against *Pseudomonas aeruginosa*, *Acinetobacter baumannii*, and *MRSA* were identified [85,93].

### 4.3. Challenges Facing the Four Databases

Due to rapid development in the field of AMPs, many databases have been established, and their comprehensiveness and accuracy are two key points determining the extent of their effect. The first key point is that in all AMP databases, the design and prediction of information of AMPs are too limited, as only antimicrobial activity (MIC) is considered as a screening index [66,67,78,79]. One single index of activity is not enough to support screening for the best candidate consistently with the expectation of targeting the AMP as a final whole. More function indexes should be included to meet the full range of requirements for various aspects of practice. Low toxicity, stability, and specificity and high yield (except for antibacterial activity) should also be considered during design and evaluation, as they are closely related to viability, persistence, precision, cost, and other factors as new candidate drugs [24]. Coordinating the above parameters and merging them in a scientifically appropriate way are major challenges to constructing or improving AMP databases but also represent an opportunity for improvement and optimization.

## 5. Performance of Database Tools for Screening

Many peptides are found by researchers in vivo and in vitro, and their antibacterial activity and stability cannot be easily ensured. Considering the high cost and labor-intensive experimental identification of AMPs, many computational methods have been proposed for prediction with different functional types and a de novo design for more new and more effective antimicrobial agents. In order to enhance the clinical application of AMPs, researchers have tended to focus exclusively on traditional rational design to increase their antibacterial activity, proteolytic resistance, and production [20]. New approaches are necessary, particularly in the field of bioinformatics, as we know that these databases are only partly used to predict and design AMPs. For example, AureinM3 and PT-5 were designed using APD, and their mutants were analyzed by APD and CAMP in 2018 and 2021, respectively [80,81]. In 2018, combined with biological information software from sequence comparison and conservative sequences in cathelicidin and aurein, Natthaports designed a series of short hybrid peptides using APD3, I-TASSER, and Expaasy and achieved impressive results [92,93,94,95,96]. These examples verify that these databases can accurately predict the reliability of AMPs, showing strong ability as a BI tool that is dependent on the scientific construction scheme of the database for the goals of mining and design.

**Figure 5:**
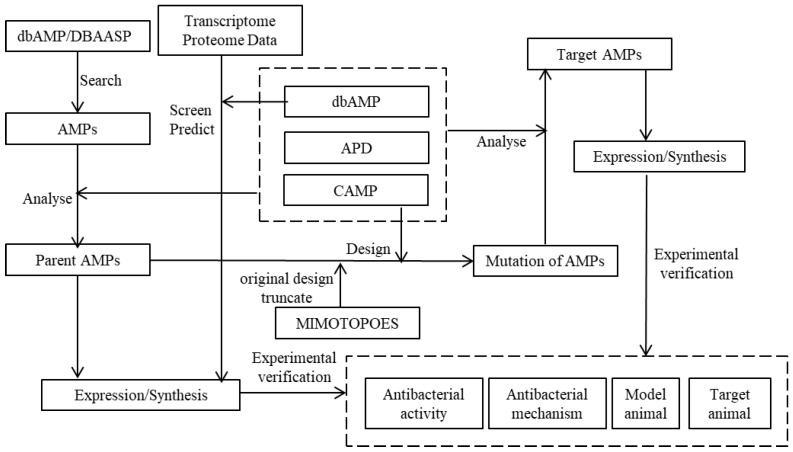
Screening and design scheme of new AMPs.

Based on the above extensive analysis of four AMP databases, an integrative approach to the design and construction of new AMP databases is proposed based on three essential key principles defined in the following three paragraphs (see Figure 5). The first key principle covers the following five points: (i) transcriptomic and proteomic data are obtained and analyzed from the dbAMP database by the AMPfinder function option; (ii) the hydrophobicity, isoelectric point, amphiphilicity, number of net charges, and other properties of the target peptide are predicted, screened, and designed by means of the AMP Calculator and predictor in the APD or the tools in the DBAASP; (iii) the druggability of candidate AMPs is analyzed and evaluated using CAMP; (iv) the key activity of AMPs on different specific target pathogenic species is predicted using the AMPpredictor and dbAMP database and then tested and screened by trials in vitro/vivo; (v) products are obtained by expression or chemical synthesis at a reasonable cost, and their bio-activity and mechanism are verified clearly by in vivo/in vitro experiments.

The second key principle also includes five points: (i) de novo design of AMPs by the DBAASP or dbAMP database with different target search parameters scheduled with convenient adjustable running and responding by choice in an option or box, such as cationic strength and specific action on different Gram-type or specific target bacteria, biofilms, or DNA and other target biomacromolecules in pathogens; (ii) the design and evaluation on hydrophobicity, isoelectric points, amphipathicity, bio-safety, stability, and other properties of target AMPs are predicted by the AMP Calculator, the predictor in the APD, and tools in the DBAASP; (iii) the AMPs odds are predicted by the CAMP; (iv) the AMPpredictor tool is used to analyze activity strength and spectrum against different species, and target candidate peptides are screened after a series of predictions and analyses; (v) candidate peptides are acquired by expression/synthesis, and the activity and mechanism are verified through in vivo/in vitro experiments.

The third principle deals with derived and modified AMPs by addressing/responding to the system through an optimization cycle to achieve the best results. The integrated scheme includes unique modules of the four AMP databases to screen and predict AMPs, which increases efficiency in designing AMPs.

## 6. Conclusions

AMPs merit more attention than they are currently receiving, and further extensive research is required, as they represent one of the pioneer ATAs with very strong potential, across multiple dimensions, to reduce stresses and threats of the AMRs in the ecosystem [8]. Due to the significant amount of AMPs with transcriptome and proteome data obtained by high-throughput technology, it is expensive and labor-intensive to carry out verification using the index of antibacterial activity alone during long chains of experiments that may go on for years [52]. Therefore, it is necessary to establish an AMP database that combines bioinformatics technology, computer algorithms, machine learning, data mining, and AI with experimental verification. Today, a number of AMP databases based on computer algorithms are in operation. They play an important role in the prediction, screening, and design of AMPs. However, difficulties exist in choosing the best one among them beyond considering antimicrobial activity. Using AMP databases, running a comprehensive analysis with high capacity and efficiency on a large data set to determine activity, toxicity, stability, specificity, and expression ability to predict AMPs could be carried out quickly by simultaneously using accurate machine learning algorithms and other new powerful BI/AI tools [52,56]. More and better new AMPs could be created quickly by means of these AMP databases and they could play an important role in the struggle to alleviate the threat posed by AMRs to the health ecosystem. We believe that the integrative approach, proposed in this paper, will lead to the improvement of AMP databases, allowing wide coverage and balance among those three essential key principles, and final goals ranging from druggability, activity, safety, stability, resistance, and cost [97].

## Figures and Tables

**Table 1:** Statistical comparison of the functional activities of four AMP libraries.

Database	APD3	CAMP3	DBAASP3	dbAMP2
Peptide source	natural/synthetic	natural/synthetic/ predict2659	natural18719/ synthetic557	natural/synthetic18345/ predict10364
Signature (family based)	-	114	-	-
Structures	447	757	400	3444
Patents	-	2083	-	-
Anti-Gram(+)	2817	2915	14,280	11,776
Anti-Gram(−)			15,139	12,191
Anti-Viral	200	117	1324	1803
Anti-Fungal	1234	1144	5620	5540
Anti-Parasitic	140	33	42	412
Anti-MRSA	206	-	-	-
Anti-Biofilm	72	-	-	36
Anti-Cancer	262	-	2917	2290
Anti-inflammatory	31	-	-	4
Anti-yeast	-	-	-	5

Note: - means not counted.

**Table 2:** Comparison of the composition and function of the four AMP libraries.

Database	APD3	CAMP3	DBAASP3	dbAMP2
Full name	Antimicrobial Peptides Database	Collection of Anti-Microbial Peptides	Database of antimicrobial activity and structure of peptides	database of Antimicrobial Peptides
Website	https://aps.unmc.edu/prediction, accessed on 4 January 2016	http://www.camp.bicnirrh.res.in/, accessed on 4 January 2016	https://www.dbaasp.org/home, accessed on 8 January 2021	http://csb.cse.yzu.edu.tw/dbAMP/, accessed on 7 January 2022
AMPs	3324	8164	18,719	26,447
Creation time	2003	2010	2014	2018
Links	NCBI, PDB, Uniprot	NCBI, PDB, APD, Uniprot	NCBI, Uniprot	NCBI, Uniprot, EMBL, PDB, APD, CAMP
Modules	21 (Calculator Predictor)	9 (Databases, Tools, search)	9 (Home, Search, Tools)	7 (Search, Analyze)
Tools	22	9	5	3
Predictive model	-	Random Forest	-	Random Forest
Multi-species detection	-	-	-	+
Antibacterial activity	-	-	-	+
NGS Sequence analysis	-	-	-	+
Anti-pathogenic activity	NC	NC	+	NC
Hemolytic/Cytotoxicity	NC	NC	+	NC
Target cell	+	-	+	+
Predict tools	-	+	+	+
AMPs design	-	+	-	-
Sequence analysis	+	-	+	-
AMPs Synthesis Types	-	-	+	-

Note: NC means not complete; + means complete; - means no.

## Data Availability

All data generated or analysed during this review are included in this published article.

## References

[1] Davies J., Davies D. (2010). Origins and evolution of antibiotic resistance. Microbiol. Mol. Biol. Rev..

[2] Gale E.F. (1947). Correlation between penicillin resistance and assimilation affinity in *Staphylococcus aureus*. Nature.

[3] Shehreen S., Chyou T.Y., Fineran P.C., Brown C.M. (2019). Genome-wide correlation analysis suggests different roles of CRISPR-Cas systems in the acquisition of antibiotic resistance genes in diverse species. Philos. Trans. R. Soc. Lond. B Biol. Sci..

[4] Huemer M., Shambat S.M., Brugger S.D., Zinkernagel A.S. (2020). Antibiotic resistance and persistence-Implications for human health and treatment perspectives. EMBO Rep..

[5] Karadag A.S., Kayıran M.A., Wu C.Y., Chen W., Parish L.C. (2021). Antibiotic resistance in acne: Changes, consequences and concerns. J. Eur. Acad. Dermatol. Venereol..

[6] Luo Y., Song Y. (2021). Mechanism of Antimicrobial Peptides: Antimicrobial, Anti-inflammatory and antibiofilm activities. Int. J. Mol. Sci..

[7] Kaschnitz R., Kreil G. (1978). Processing of prepromelittin by subcellular fractions from rat liver. Biochem. Biophys. Res. Commun..

[8] Fennell J.F., Shipman W.H., Cole L.J. (1967). Antibacterial Action of a Bee Venom Fraction (Melittin) against a Penicillin-Resistant Staphylococcus and Other Microorganisms.

[9] Boman H.G., Nilsson I., Rasmuson B. (1972). Inducible antibacterial defence system in Drosophila. Nature.

[10] Hultmark D., Engstrom A., Bennich H., Kapur R., Boman H.G. (1982). Insect immunity: Isolation and structure of cecropin D and four minor antibacterial components from *Cecropiapupae*. Eur. J. Biochem..

[11] Qu Z., Steiner H., Engstrom A., Bennich H., Boman H.G. (1982). Insect immunity: Isolation and structure of cecropins B and D from pupae of the Chinese oak silk moth, *Antheraeapernyi*. Eur. J. Biochem..

[12] Boman H.G., Faye I., von Hofsten P., Kockum K., Lee J.Y., Xanthopoulos K.G., Bennich H., Engstrom A., Merrifield R.B., Andreu D. (1985). On the primary structures of lysozyme, cecropins and attacins from *Hyalophoracecropia*. Dev. Comp. Immunol..

[13] Lidholm D.A., Gudmundsson G.H., Xanthopoulos K.G., Boman H.G. (1987). Insect immunity: cDNA clones coding for the precursor forms of cecropins A and D, antibacterial proteins from *Hyalophoracecropia*. FEBS Lett..

[14] Hou F., Li J., Pan P., Xu J., Liu L., Liu W., Song B., Li N., Wan J., Gao H. (2011). Isolation and characterisation of a new antimicrobial peptide from the skin of *Xenopuslaevis*. Int. J. Antimicrob. Agents.

[15] Kumar N.S.S., Nazeer R.A., Jaiganesh R. (2012). Purification and identification of antioxidant peptides from the skin protein hydrolysate of two marine fishes, horse mackerel (*Magalaspiscordyla*) and croaker (*Otolithesruber*). Amino Acids.

[16] Elsbach P., Weiss J., Levy O. (1994). Integration of antimicrobial host defenses: Role of the bactericidal/permeability-increasing protein. Trends Microbiol..

[17] Ramamoorthy A., Thennarasu S., Lee D.K., Tan A., Maloy L. (2006). Solid-state NMR investigation of the membrane-disrupting mechanism of antimicrobial peptides MSI-78 and MSI-594 derived from magainin 2 and melittin. Biophys. J..

[18] Mor A., Nicolas P. (1994). Isolation and structure of novel defensive peptides from frog skin. Eur. J. Biochem..

[19] Levy O. (2000). Antimicrobial proteins and peptides of blood: Templates for novel antimicrobial agents. Blood.

[20] Gong Z., Pei X., Ren S., Chen X., Wang L., Ma C., Xi X., Chen T., Shaw C., Zhou M. (2020). Identification and rational design of a novel antibacterial peptide dermaseptin-AC from the skin secretion of the red-eyed tree frog *Agalychnis callidryas*. Antibiotics.

[21] Pasupuleti M., Schmidtchen A., Malmsten M. (2012). Antimicrobial peptides: Key components of the innate immune system. Crit. Rev. Biotechnol..

[22] Kumar P., Kizhakkedathu J.N., Straus S.K. (2018). Antimicrobial Peptides: Diversity, mechanism of action and strategies to improve the activity and biocompatibility in vivo. Biomolecules.

[23] Lai R., Zheng Y.T., Shen J.H., Liu G.J., Liu H., Lee W.H., Tang S.Z., Zhang Y. (2002). Antimicrobial peptides from skin secretions of Chinese red belly toad *Bombina maxima*. Peptides.

[24] Cao X., Zhang Y., Mao R., Teng D., Wang X., Wang J. (2015). Design and recombination expression of a novel plectasin-derived peptide MP1106 and its properties against *Staphylococcus aureus*. Appl. Microbiol. Biotechnol..

[25] Slaninová J., Mlsová V., Kroupová H., Alán L., Tůmová T., Monincová L., Borovičková L., Fučík V., Ceřovský V. (2012). Toxicity study of antimicrobial peptides from wild bee venom and their analogs toward mammalian normal and cancer cells. Peptides.

[26] Zheng X., Yang N., Mao R., Hao Y., Teng D., Wang J. (2022). Pharmacokinetics and pharmacodynamics of fungal defensin NZX against *Staphylococcus aureus*-Induced mouse peritonitis model. Front. Microbiol..

[27] Liu H., Yang N., Teng D., Mao R., Hao Y., Ma X., Wang J. (2021). Design and pharmacodynamics of recombinant fungus defensin NZL with improved activity against *Staphylococcus hyicus* In Vitro and In Vivo. Int. J. Mol. Sci..

[28] Hao Y., Wang J., de la Fuente-Nunez C., Franco O.L. (2022). Editorial: Antimicrobial Peptides: Molecular design, structure-function relationship, and biosynthesis optimization. Front. Microbiol..

[29] Wu Y., Yang N., Mao R., Hao Y., Teng D., Wang J. (2022). In vitro pharmacodynamics and bactericidal mechanism of fungal defensin-derived peptides NZX and P2 against *Streptococcus agalactiae*. Microorganisms.

[30] Ma X., Yang N., Mao R., Hao Y., Yan X., Teng D., Wang J. (2021). The pharmacodynamics study of insect defensin DLP4 against toxigenic *Staphylococcus hyicus* ACCC 61734 in Vitro and Vivo. Front. Cell. Infect. Microbiol..

[31] Yang N., Teng D., Mao R., Hao Y., Wang X., Wang Z., Wang X., Wang J. (2019). A recombinant fungal defensin-like peptide-P2 combats multidrug-resistant *Staphylococcus aureus* and biofilms. Appl. Microbiol. Biotechnol..

[32] Lee S.B., Li B., Jin S., Daniell H. (2011). Expression and characterization of antimicrobial peptides Retrocyclin-101 and Protegrin-1 in chloroplasts to control viral and bacterial infections. Plant Biotechnol. J..

[33] Bellamy W., Takase M., Wakabayashi H., Kawase K., Tomita M. (1992). Antibacterial spectrum of lactoferricin B, a potent bactericidal peptide derived from the N-terminal region of bovine lactoferrin. J. Appl. Bacteriol..

[34] Hastings J.W., Sailer M., Johnson K., Roy K.L., Vederas J.C., Stiles M.E. (1991). Characterization of leucocin A-UAL 187 and cloning of the bacteriocin gene from *Leuconostoc gelidum*. J. Bacteriol..

[35] Bals R. (2000). Epithelial antimicrobial peptides in host defense against infection. Respir. Res..

[36] Selsted M.E., Novotny M.J., Morris W.L., Tang Y.Q., Smith W., Cullor J.S. (1992). Indolicidin, a novel bactericidal tridecapeptide amide from neutrophils. J. Biol. Chem..

[37] Nguyen L.T., Haney E.F., Vogel H.J. (2011). The expanding scope of antimicrobial peptide structures and their modes of action. Trends Biotechnol..

[38] Silverstein K.A., Moskal W.A., Wu H.C., Underwood B.A., Graham M.A., Town C.D., VandenBosch K.A. (2007). Small cysteine-rich peptides resembling antimicrobial peptides have been under-predicted in plants. Plant J..

[39] Resende J.M., Moraes C.M., Prates M.V., Cesar A., Almeida F.C., Mundim N.C., Valente A.P., Bemquerer M.P., Piló-Veloso D., Bechinger B. (2008). Solution NMR structures of the antimicrobial peptides phylloseptin-1, -2, and -3 and biological activity: The role of charges and hydrogen bonding interactions in stabilizing helix conformations. Peptides.

[40] Sakagami-Yasui Y., Shirafuji Y., Yamasaki O., Morizane S., Hamada T., Umemura H., Iwatsuki K. (2017). Two arginine residues in the COOH-terminal of human β-defensin-3 constitute an essential motif for antimicrobial activity and IL-6 production. Exp. Dermatol..

[41] Zhu Y., Akhtar M.U., Li B., Chou S., Shao C., Li J., Shan A. (2022). The design of cell-selective tryptophan and arginine-rich antimicrobial peptides by introducing hydrophilic uncharged residues. Acta Biomater..

[42] Jin L., Bai X., Luan N., Yao H., Zhang Z., Liu W., Chen Y., Yan X., Rong M., Lai R. (2016). A designed tryptophan- and lysine/arginine-rich antimicrobial peptide with therapeutic potential for clinical antibiotic-resistant *Candida albicans* vaginitis. J. Med. Chem..

[43] Saravanan R., Li X., Lim K., Mohanram H., Peng L., Mishra B., Basu A., Lee J.M., Bhattacharjya S., Leong S.S. (2014). Design of short membrane selective antimicrobial peptides containing tryptophan and arginine residues for improved activity, salt-resistance, and biocompatibility. Biotechnol. Bioeng..

[44] Svenson J., Karstad R., Flaten G.E., Brandsdal B.O., Brandl M., Svendsen J.S. (2009). Altered activity and physicochemical properties of short cationic antimicrobial peptides by incorporation of arginine analogues. Mol. Pharm..

[45] Panteleev P.V., Bolosov I.A., Balandin S.V., Ovchinnikova T.V. (2015). Design of antimicrobial peptide arenicin analogs with improved therapeutic indices. J. Pept. Sci..

[46] Brogden K.A. (2005). Antimicrobial peptides: Pore formers or metabolic inhibitors in bacteria?. Nat. Rev. Microbiol..

[47] Rončević T., Gerdol M., Mardirossian M., Maleš M., Cvjetan S., Benincasa M., Maravić A., Gajski G., Krce L., Aviani I. (2022). Anisaxins, helical antimicrobial peptides from marine parasites, kill resistant bacteria by lipid extraction and membrane disruption. Acta Biomater..

[48] Sychev S.V., Balandin S.V., Panteleev P.V., Barsukov L.I., Ovchinnikova T.V. (2015). Lipid-dependent pore formation by antimicrobial peptides arenicin-2 and melittin demonstrated by their proton transfer activity. J. Pept. Sci..

[49] Teixeira V., Feio M.J., Bastos M. (2012). Role of lipids in the interaction of antimicrobial peptides with membranes. Prog. Lipid Res..

[50] Mygind P.H., Fischer R.L., Schnorr K.M., Hansen M.T., Sönksen C.P., Ludvigsen S., Raventós D., Buskov S., Christensen B., De Maria L. (2005). Plectasin is a peptide antibiotic with therapeutic potential from a saprophytic fungus. Nature.

[51] Lazzaro B.P., Zasloff M., Rolff J. (2020). Antimicrobial peptides: Application informed by evolution. Science.

[52] Nagarajan D., Nagarajan T., Roy N., Kulkarni O., Ravichandran S., Mishra M., Chakravortty D., Chandra N. (2018). Computational antimicrobial peptide design and evaluation against multidrug-resistant clinical isolates of bacteria. J. Biol. Chem..

[53] Porto W.F., Pires A.S., Franco O.L. (2017). Computational tools for exploring sequence databases as a resource for antimicrobial peptides. Biotechnol. Adv..

[54] Wang G., Zietz C.M., Mudgapalli A., Wang S., Wang Z. (2022). The evolution of the antimicrobial peptide database over 18 years: Milestones and new features. Protein Sci..

[55] Chakraborty S., Phu M., de Morais T.P., Nascimento R., Goulart L.R., Rao B.J., Asgeirsson B., Dandekar A.M. (2014). The PDB database is a rich source of alpha-helical anti-microbial peptides to combat disease causing pathogens. F1000 Res..

[56] Plisson F., Ramírez-Sánchez O., Martínez-Hernández C. (2020). Machine learning-guided discovery and design of non-hemolytic peptides. Sci Rep..

[57] Brahmachary M., Krishnan S.P., Koh J.L., Khan A.M., Seah S.H., Tan T.W., Brusic V., Bajic V.B. (2004). ANTIMIC: A database of antimicrobial sequences. Nucleic Acids Res..

[58] Hammami R., Ben Hamida J., Vergoten G., Fliss I. (2009). PhytAMP: A database dedicated to antimicrobial plant peptides. Nucleic Acids Res..

[59] Shi G., Kang X., Dong F., Liu Y., Zhu N., Hu Y., Xu H., Lao X., Zheng H. (2022). DRAMP 3.0: An enhanced comprehensive data repository of antimicrobial peptides. Nucleic Acids Res..

[60] Gabere M.N., Noble W.S. (2017). Empirical comparison of web-based antimicrobial peptide prediction tools. Bioinformatics.

[61] Wang G. (2015). Improved methods for classification, prediction, and design of antimicrobial peptides. Methods Mol. Biol..

[62] Thomas S., Karnik S., Barai R.S., Jayaraman V.K., Idicula-Thomas S. (2010). CAMP: A useful resource for research on antimicrobial peptides. Nucleic Acids Res..

[63] Pirtskhalava M., Gabrielian A., Cruz P., Griggs H.L., Squires R.B., Hurt D.E. (2016). DBAASP v.2: An enhanced database of structure and antimicrobial/cytotoxic activity of natural and synthetic peptides. Nucleic Acids Res..

[64] Jhong J.H., Chi Y.H., Li W.C., Lin T.H., Huang K.Y., Lee T.Y. (2019). dbAMP: An integrated resource for exploring antimicrobial peptides with functional activities and physicochemical properties on transcriptome and proteome data. Nucleic Acids Res..

[65] Jhong J.H., Yao L., Pang Y., Li Z., Chung C.R., Wang R., Li S., Li W., Luo M., Ma R. (2022). dbAMP 2.0: Updated resource for antimicrobial peptides with an enhanced scanning method for genomic and proteomic data. Nucleic Acids Res..

[66] Pirtskhalava M., Amstrong A.A., Grigolava M., Chubinidze M., Alimbarashvili E., Vishnepolsky B., Gabrielian A., Rosenthal A., Hurt D.E., Tartakovsky M. (2021). DBAASP v3: Database of antimicrobial/cytotoxic activity and structure of peptides as a resource for development of new therapeutics. Nucleic Acids Res..

[67] Wang G., Li X., Wang Z. (2016). APD3: The antimicrobial peptide database as a tool for research and education. Nucleic Acids Res..

[68] Lee J., Kang H.K., Cheong H., Park Y. (2021). A novel antimicrobial peptides from pine needles of *Pinus densiflora Sieb. Et Zucc.* against foodborne bacteria. Front. Microbiol..

[69] Dean S.N., Alvarez J.A.E., Zabetakis D., Walper S.A., Malanoski A.P. (2021). PepVAE: Variational autoencoder framework for antimicrobial peptide generation and activity prediction. Front. Microbiol..

[70] Gu J., Isozumi N., Yuan S., Jin L., Gao B., Ohki S., Zhu S. (2021). Evolution-based protein engineering for antifungal peptide improvement. Mol. Biol. Evol..

[71] Yang Y., Gao H., Liu W., Liu X., Jiang X., Li X., Wu Q., Xu Z., Zhao Q. (2021). *Arctiumlappa* L. roots ameliorates cerebral ischemia through inhibiting neuronal apoptosis and suppressing AMPK/mTOR-mediated autophagy. Phytomedicine.

[72] Li Y., Li G.X., Yu M.L., Liu C.L., Qu Y.T., Wu H. (2021). Association between anxiety symptoms and problematic smartphone use among chinese university students: The mediating/moderating role of self-efficacy. Front. Psychiatry.

[73] Mao R., Teng D., Wang X., Xi D., Zhang Y., Hu X., Yang Y., Wang J. (2013). Design, expression, and characterization of a novel targeted plectasin against methicillin-resistant *Staphylococcus aureus*. Appl. Microbiol. Biotechnol..

[74] Zavala-Soto J.O., Hernandez-Rivero L., Tapia-Fonllem C. (2022). Pro-lactation cesarean section: Immediate skin-to-skin contact and its influence on prolonged breastfeeding. Front. Sociol..

[75] Moreno-Barahona M., Fraijo-Sing B., Fleury-Bahi G., Navarro-Carrascal O., Tapia-Fonllem C. (2022). Conceptual integration and empirical validation of a unified taxonomy: Quantitative data analysis for virtual learning environments. Front. Psychol..

[76] Wang G., Li X., Wang Z. (2009). APD2: The updated antimicrobial peptide database and its application in peptide design. Nucleic Acids Res..

[77] Waghu F.H., Gopi L., Barai R.S., Ramteke P., Nizami B., Idicula-Thomas S. (2014). CAMP: Collection of sequences and structures of antimicrobial peptides. Nucleic Acids Res..

[78] Waghu F.H., Idicula-Thomas S. (2020). Collection of antimicrobial peptides database and its derivatives: Applications and beyond. Protein Sci..

[79] Fan L., Sun J., Zhou M., Zhou J., Lao X., Zheng H., Xu H. (2016). DRAMP: A comprehensive data repository of antimicrobial peptides. Sci. Rep..

[80] Memariani H., Shahbazzadeh D., Ranjbar R., Behdani M., Memariani M., Bagheri K.P. (2017). Design and characterization of short hybrid antimicrobial peptides from pEM-2, mastoparan-VT1, and mastoparan-B. Chem. Biol. Drug Des..

[81] Chen X., Yi Y., You X., Liu J., Shi Q. (2019). High-Throughput identification of putative antimicrobial peptides from multi-omics data of the lined seahorse (*Hippocampus erectus*). Mar. Drugs.

[82] Sychev S.V., Sukhanov S.V., Panteleev P.V., Shenkarev Z.O., Ovchinnikova T.V. (2017). Marine antimicrobial peptide arenicin adopts a monomeric twisted β-hairpin structure and forms low conductivity pores in zwitterionic lipid bilayers. Biopolymers.

[83] Wang Z., Yang N., Teng D., Hao Y., Li T., Han H., Mao R., Wang J. (2022). Resistance response to Arenicin derivatives in *Escherichia coli*. Appl. Microbiol. Biotechnol..

[84] Ricardo F., Pradilla D., Cruz J.C., Alvarez O. (2021). Emerging Emulsifiers: Conceptual Basis for the identification and iational design of peptides with surface activity. Int. J. Mol. Sci..

[85] Capecchi A., Cai X., Personne H., Köhler T., van Delden C., Reymond J.L. (2021). Machine learning designs non-hemolytic antimicrobial peptides. Chem. Sci..

[86] Wang G., Vaisman I.I., van Hoek M.L. (2022). Machine Learning Prediction of Antimicrobial Peptides. Computational Peptide Science.

[87] Okella H., Georrge J.J., Ochwo S., Ndekezi C., Koffi K.T., Aber J., Ajayi C.O., Fofana F.G., Ikiriza H., Mtewa A.G. (2020). New putative antimicrobial candidates: In silico design of fish-derived antibacterial peptide-motifs. Front. Bioeng. Biotechnol..

[88] Xu J., Li F., Leier A., Xiang D., Shen H.H., Marquez Lago T.T., Li J., Yu D.J., Song J. (2021). Comprehensive assessment of machine learning-based methods for predicting antimicrobial peptides. Brief Bioinform..

[89] Vishnepolsky B., Pirtskhalava M. (2019). Comment on: ‘Empirical comparison of web-based antimicrobial peptide prediction tools. Bioinformatics.

[90] Lata S., Mishra N.K., Raghava G.P. (2010). AntiBP2: Improved version of antibacterial peptide prediction. BMC Bioinform..

[91] Wang G. (2013). Database-Guided Discovery of Potent Peptides to Combat *HIV-1* or Superbugs. Pharmaceuticals.

[92] Dong Y., Lushnikova T., Golla R.M., Wang X., Wang G. (2017). Small molecule mimics of DFTamP1, a database designed anti-*Staphylococcal* peptide. Bioorg. Med. Chem..

[93] Moretta A., Salvia R., Scieuzo C., Di Somma A., Vogel H., Pucci P., Sgambato A., Wolff M., Falabella P. (2020). A bioinformatic study of antimicrobial peptides identified in the Black Soldier Fly (BSF) *Hermetiaillucens* (Diptera: Stratiomyidae). Sci. Rep..

[94] Majumder A., Biswal M.R., Prakash M.K. (2019). Computational screening of antimicrobial peptides for *Acinetobacter baumannii*. PLoS ONE.

[95] Houyvet B., Zanuttini B., Corre E., Le Corguillé G., Henry J., Zatylny-Gaudin C. (2018). Design of antimicrobial peptides from a cuttlefish database. Amino Acids.

[96] Ramezanzadeh M., Saeedi N., Mesbahfar E., Farrokh P., Salimi F., Rezaei A. (2021). Design and characterization of new antimicrobial peptides derived from aurein 1.2 with enhanced antibacterial activity. Biochimie.

[97] Yang N., Aminov R., Franco O.L., de la Fuente-Nunez C., Wang J. (2023). Editorial: Community series in antimicrobial peptides: Molecular design, structure function relationship and biosynthesis optimization. Front. Microbiol..

